# Conventional Ultrasound and Contrast-Enhanced Ultrasound in Hepatic Epithelioid Hemangioendothelioma: Retrospective Evaluation in 20 Cases

**DOI:** 10.3389/fonc.2022.686650

**Published:** 2022-02-28

**Authors:** Tingting Qiu, Dongmei Zhu, Rong Fu, Yan Luo, Wenwu Ling

**Affiliations:** ^1^Department of Medical Ultrasound, West China Hospital of Sichuan University, Chengdu, China; ^2^Department of Ultrasound, The Second Clinical Medical College of Jinan University (Shenzhen People’s Hospital), Shenzhen, China; ^3^Department of Ultrasound, The Affiliated Nanchong Central Hospital of North Sichuan Medical College, Nanchong, China

**Keywords:** conventional ultrasound, contrast-enhanced ultrasound, hepatic epithelioid hemangioendothelioma, liver, diagnosis

## Abstract

**Objectives:**

This study aimed to analyze the patterns of conventional ultrasound (CUS) and contrast-enhanced ultrasound (CEUS) in 20 patients with diagnosis of hepatic epithelioid hemangioendothelioma (HEHE).

**Methods:**

Twenty patients (12 females and 8 males) with mean age of 43.6 ± 13.6 years were included in this study from January 2012 to May 2020. CUS, CEUS, computed tomography (CT) and magnetic resonance imaging (MRI) features of the twenty patients with histologically proven HEHE were retrospectively reviewed by two radiologists. The clinical manifestations and the pathological findings of all patients with HEHE are described.

**Results:**

There were 3 types of HEHE in imaging, including single nodular (8/20, 40%), multifocal nodular (10/20, 50%), and diffuse type (2/20, 10%). The mean size of lesions was 4.2 ± 2.6 cm. B-mode ultrasound of HEHE showed hypoechoic (15/20, 75%), heterogeneous echogenicity (4/20, 20%), or hyperechoic (1/20, 5%) lesions with regular shape (18/20, 90%) near the liver capsule (17/20, 85%), and occasionally with a halo (4/20, 20%) and calcifications (3/20, 15%). Eight out of the 20 patients also had CEUS. On CEUS, HEHE demonstrated peripheral rim-like (5/8, 62.5%), heterogeneous (2/8, 25%), or homogeneous (1/8, 12.5%) hyperenhancement in the arterial phase. All patients (8/8, 100%) showed hypoenhancement in the portal and late phase. CEUS detected more lesions than CUS in 3 patients (3/8, 37.5%). In addition, central irregular unenhanced zones were observed in 6 patients (6/8, 75%). On contrast-enhanced CT or MRI, most cases presented with capsule retraction sign and lollipop sign.

**Conclusions:**

HEHE demonstrated specific findings on ultrasound, which includes multifocal hypoechoic lesions in a subcapsular distribution with typical enhancement characteristics of malignant hepatic tumors.

## Introduction

Hepatic epithelioid hemangioendothelioma (HEHE) is a very rare malignant tumor of vascular origin, with biological manifestation between those of hemangioma and angiosarcoma (1). It was first described by Ishak et al. in 1984, with an incidence of 0.1-0.2 per in100,000 population, accounting for 1% of all primary hepatic malignant neoplasm (2). The clinical manifestations of HEHE are very variable, ranging from asymptomatic to portal hypertension or hepatic failure. The most common manifestations are nonspecific, including right upper quadrant pain and weight loss (3, 4). HEHE usually has an indolent clinical course, and long-term survival after liver tumor resection is good (2, 3, 5). However, due to the rarity of HEHE and its nonspecific clinical manifestations, the definitive diagnosis of HEHE is very challenging for radiologists.

Imaging modalities has an important role for preoperative detection and diagnosis of liver tumors, therefore, recognition of the imaging findings of HEHE may be beneficial for its detection and diagnosis. HEHE is often incidentally found by routine imaging with computed tomography (CT), magnetic resonance imaging (MRI) or ultrasound. However, only a few papers have presented the imaging study of HEHE, and most of them were case reports or studies with very small sample size. In addition, these studies mainly used CT or MRI (6–8), conventional ultrasound (CUS) and contrast enhanced ultrasound (CEUS) features of HEHE have rarely been reported (9–12). In current clinical practice, CUS is usually the first line imaging modality used for evaluation of focal liver lesions (FLLs), and CEUS have been widely used in detection and characterization of FLLs (13). Therefore, the purpose of this study is to describe the CUS and CEUS features of histologically proven HEHE to help radiologists recognize HEHE.

## Materials and Methods

### Patients

We retrospectively analyzed the results of CUS and CEUS examination of 20 patients with histologically proven HEHE who were admitted to our hospital from January 2012 to May 2020, including twelve females and eight males with mean age of 43.6 ± 13.6 years (ranging from 23 to 69 years), and the same time, the CT and MRI image features were also briefly summarized. This study was approved by the Ethical Committee of West China Hospital of Sichuan University and written informed consent was waived. Patients gave their permission to be included in the study. In this study, all patients were confirmed histologically by US-guided percutaneous 18-gauge core needle biopsy or hepatic tumor resection.

### Ultrasound Examination

The CUS and CEUS were performed with two ultrasound systems, including the Philips IU22 scanner (Philips Medical Solutions, Mountain View) with a C5-1 convex transducer (1-5MHz) and LOGIQ E9 (GE Healthcare) with a C1-5 convex transducer (1-5MHz). After CUS, eight patients received additional CEUS, which was performed using pulse inversion harmonic real time imaging at a low mechanical index (Philips IU22, MI, 0.05). The contrast agent used was SonoVue (Braco Spa). A dose of 1.2 mL SonoVue was applied as bolus injection *via* a 20-gauge intravenous needle placed in the antecubital vein, followed by 5 mL 0.9% saline solution flush. The timer was started when the SonoVue injection was completed. Each examination was observed continuously for 5 minutes after the contrast agent injection, including the target tumor and surrounding liver parenchyma. According to well established guidelines [13], arterial phase was defined as 10-30 s after contrast injection, the portal phase was 30-120 s, and the late phase was 121-360 s. All examinations were digitally recorded on the ultrasound system.

### CT/MRI Examination

CT examination was performed with Siemens Somatom Definition FLASH scanner (Siemens, Germany), with contrast agent Iohexol Injection (300 mg/mL, dose 1.5mL/kg).

MRI examination was conducted with Siemens 3.0T trio class scanner (Siemens AG, Germany), and contrast agent was Gadolinium diethylenetriaminepenta-acetic acid (Gd-DTPA, Bayer Schering Pharm AG, Germany, dose 0.2 mmol/kg).

Each contrast-enhanced examination was recorded at 25s, 75s and 120s after contrast injection, corresponding to the arterial phase, portal phase and delayed phase.

### Image Analysis

All images were reviewed by two independent radiologists (W. Ling, and Y. Luo) who had > 5 years of experience of liver neoplasms and blinded to the clinical data and pathological results. Discordance between the radiologists was resolved by consensus.

General imaging features of HEHE included location, number of nodules (single, multiple, diffuse), maximum diameter, shape (regular or irregular), borders (well- or ill-defined), and calcifications.

CUS features included echogenicity (hypoechoic, hyperechoic, isoechoic, homogeneous or heterogeneous as compared with surrounding liver parenchyma), and the presence or absence of a peripheral hypoechoic or hyperechoic halo, and color Doppler signal.

The enhancement level and patterns of the lesions were also evaluated in different phase of imaging. According to the enhancement level of the tumor in comparison to surrounding liver parenchyma, the contrast enhancement level was divided into hypoenhancement, hyperenhancement, isoenhancement, and nonenhancement. The patterns of enhancement included homogeneous, heterogeneous, and peripheral rim-like enhancement.

### Pathological Examination

Histological specimens of tumor were obtained by US-guided percutaneous core needle biopsy (in 5 patients) or by hepatic tumor resection and nodule biopsy (in 15 patients). HEHE was confirmed histologically on hematoxylin and eosin (H&E) staining and immunohistochemical staining of tissue specimens. The endothelial origin was verified by detection of endothelial markers [CD31, CD34, and factor VIII-related antigen (FVIII Ag)] in the specimens by immunohistochemical staining. All specimens of HEHE were analyzed by two experienced pathologists (>10 years of experience of liver pathology) through consensus to improve diagnostic accuracy.

## Results

### Clinical and Laboratory Data

Among the 20 cases, twelve patients (12/20, 60%) without any complains had an incidental finding of liver tumors in a routine physical examination, five (5/20, 25%) patients suffered from right upper quadrant pain at the first presentation, three patients (3/20, 15%) presented with weight loss. The laboratory tests showed that the liver functions of all patients were within normal ranges. The serum hepatitis B virus surface antigen (HBsAg) was positive in four patients (4/20, 20%), anti-hepatitis C virus antibody was negative in all patients. The serum tumor marker alpha-fetoprotein (AFP) and carcinoembryonic antigen (CEA) were slightly elevated in two patients (2/20, 10%) and 6 patients (6/20, 30%), respectively. Cancer antigen 19-9 (CA 19-9) was increased in 6 patients (6/20, 30%) and cancer antigen 125 (CA 125) was elevated in 4 patients (4/20, 20%) (**Table 1**).

**Table 1:** The characteristics of clinical and laboratory data in 20 patients.

Characteristics of 20 Patients	
Age (yr)	
Mean±SD	43.6 ± 13.6
Range	23-69
Sex	
Male	8 (40%)
Female	12 (60%)
Symptoms	
No symptoms	12 (60%)
Right upper quadrant pain	5 (25%)
Weight loss	3 (15%)
HBsAg positive	4 (20%)
Increased serum tumor markers	
AFP	2 (10%)
CEA	6 (30%)
CA 19-9	6 (30%)
CA 125	4 (20%)

HBsAg, hepatitis B virus surface antigen; AFP, alpha-fetoprotein ; CEA, carcinoembryonic antigen; CA19-9, cancer antigen 19-9; CA125, cancer antigen 125.

### General Imaging Findings

There were three types of HEHE in our study: single nodule or mass (8/20, 40%), multifocal nodule (10/20, 50%), and diffuse subtype (2/20, 10%). In the single nodular cases, the lesions involved the right lobe of liver in 6 patients (6/8, 75%) and the left lobe of liver in 2 patients (2/8, 25%); in the multifocal nodular cases, the lesions located in both left and right hepatic lobes in 9 patients (9/10, 90%), and in the right lobe of 1 patient (1/10, 10%). In 17 patients (17/20, 85%), the majority of lesions were close to the liver capsule, and 15 patients (15/20, 75%) of them were accompanied by the capsule retraction sign. The mean size of the lesions was 4.2 ± 2.6 cm (ranging from 1.2-11.2 cm). HEHE lesions with calcifications were observed in 3 patients (3/20, 15%). Lesions with ill-defined margins were seen in 9 patients (9/20, 45%), while 11 patients showed lesions with well-defined margins (11/20, 55%). The shape of lesions was regular (round or oval) in 18 patients (18/20, 90%) and irregular in 2 patients (2/20, 10%, the diameter of the irregular lesions was more than 10 cm).

### CUS Findings

**Figure 1:**
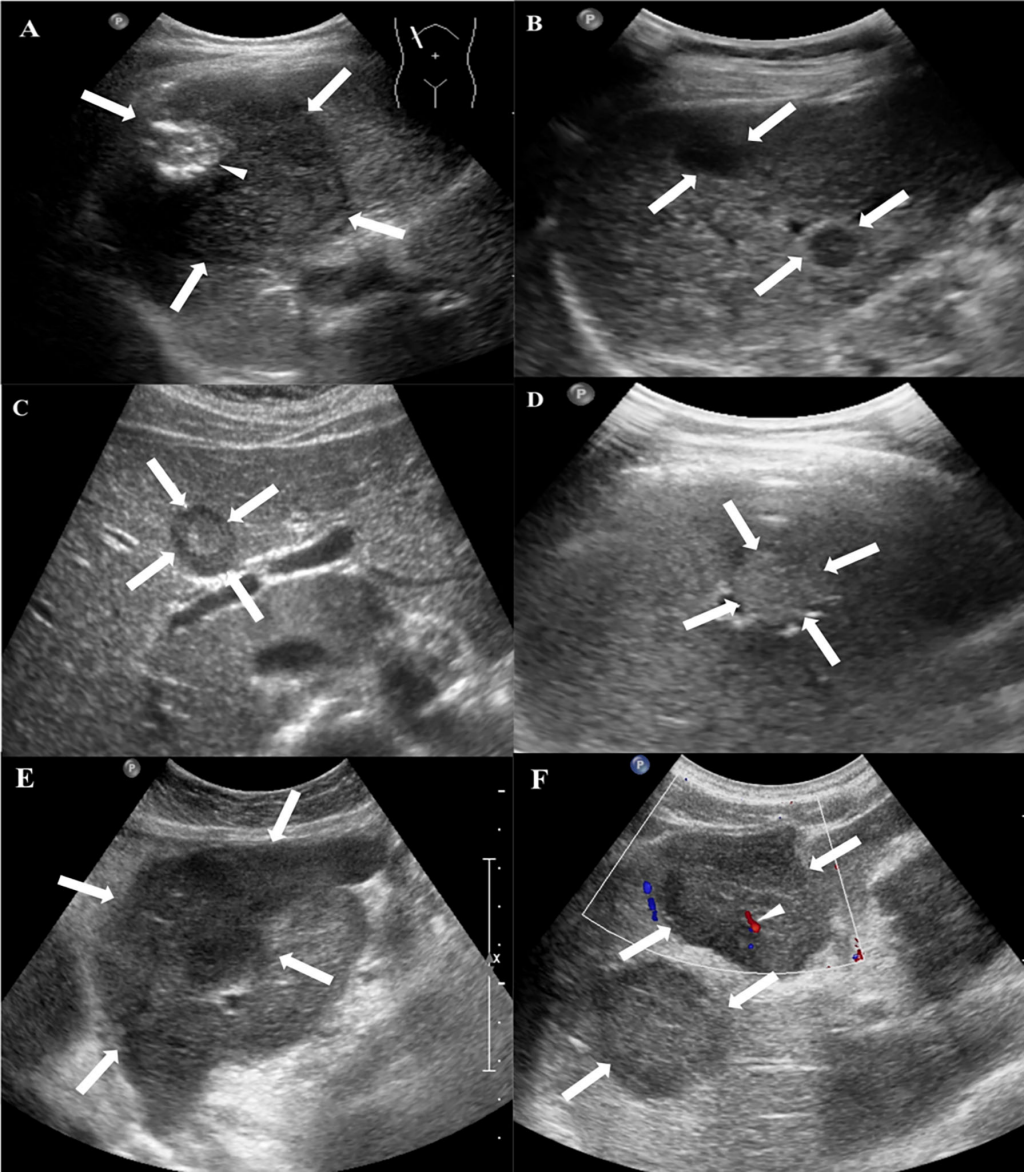
Conventional US features of HEHE in patients. **(A)** Grayscale ultrasound displayed a subcapsular hypoechoic mass (arrows) with well-defined margin, regular shape and focal calcification (small arrow) in a 49-year-old female. **(B)** Multifocal hypoechoic nodules (arrows) with a hyperechoic halo in a 48-year-old female. **(C)** A heterogeneous nodule with both hypo- and hyperechoic regions (arrows) in a 29-year-old female. **(D)** A hyperechoic nodule (arrows) with ill-defined margin in a 49-year-old male. **(E)** A hypoechoic mass with irregular shape and ill-defined margin in a 56-year-old female. **(F)** CDFI showed rod-like blood-flow signal (small arrow) in a multiple hypoechoic masses (arrows) in a 58-year-old male.

All patients received the CUS examination. Hypoechoic lesions were seen in 15 patients (15/20, 75%), heterogeneous lesions in 4 patients (4/20, 20%), and a hyperechoic lesion in one patient with single nodule (1/20, 5%). A hyperechoic or hypoechoic halo was demonstrated in 4 patients (4/20, 20%). Color Doppler flow imaging (CDFI) detected punctate or short rod-like blood-flow signals in the lesions of 6 patients (6/20, 30%) (**Figure 1**, **Table 2**).

**Table 2:** The US features of HEHE in 20 patients.

US features	
Number of lesions	
Single	8 (40%)
Multifocal	10 (50%)
Diffuse	2 (10%)
Size of lesions (cm)	
Mean±SD	4.2±2.6
Range	1.2-11.2
Location of lesions	
Left hepatic lobe	2 (10%)
Right hepatic lobe	7 (35%)
Two lobes	11 (55%)
Lesions near the liver capsule	
Yes	17 (85%)
No	3 (15%)
Echogenicity of lesions	
Hyperechoic	1 (5%)
Hypoechoic	15 (75%)
Heterogeneous echogenicity	4 (20%)
Lesions with calcification	3 (15%)
Lesions with a halo	4 (20%)
Shapes of lesions	
Regular (round or oval)	18 (90%)
Irregular	2 (10%)
Margins of lesions	
Well-defined	11 (55%)
Ill-defined	9 (45%)
Color Doppler signal in the lesions	
Yes	6 (30%)
No	14 (70%)

### CEUS Findings

**Figure 2:**
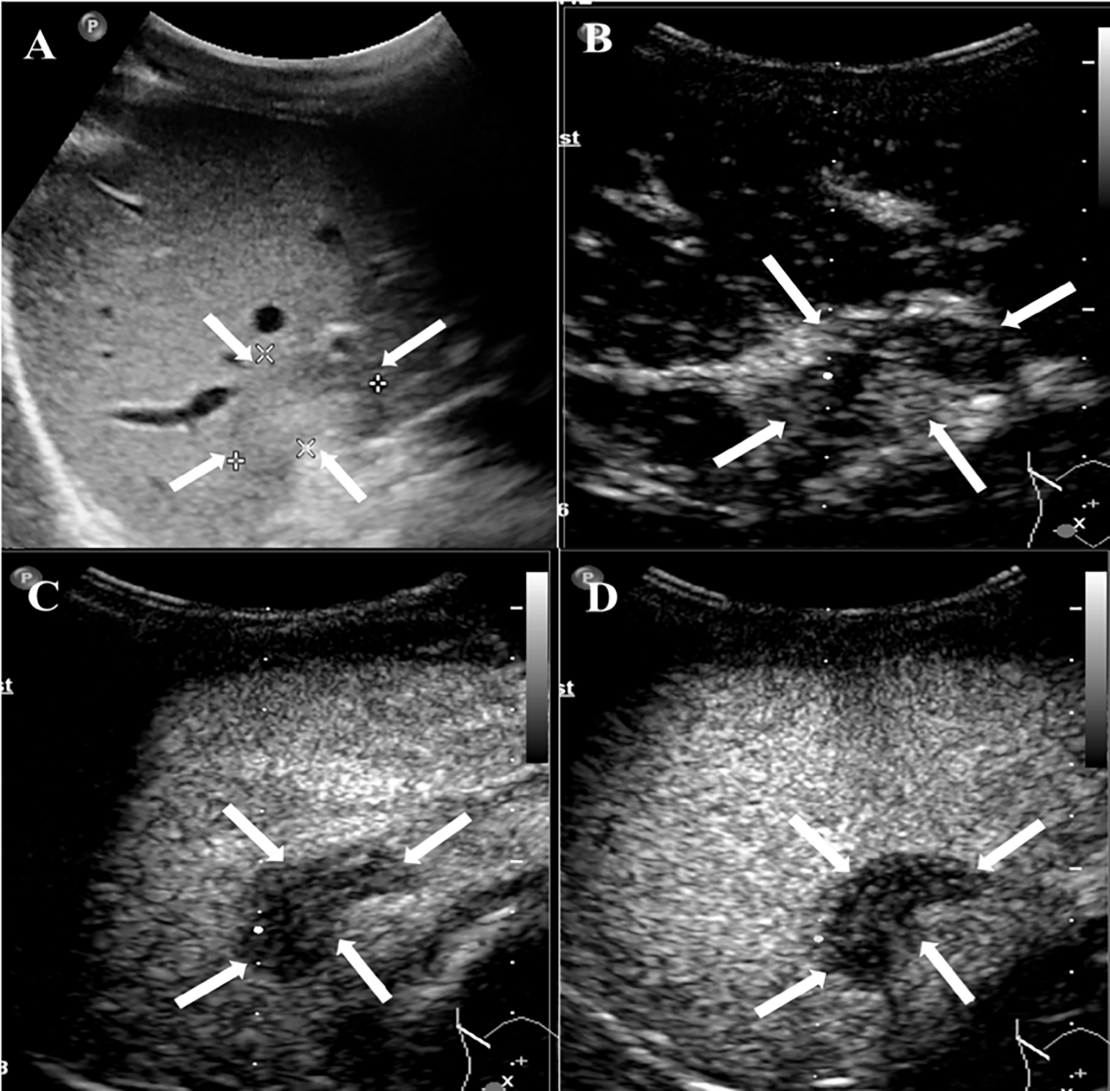
CEUS features of HEHE in a 24-year-old male. **(A)** Greyscale ultrasound presented a subcapsular hypoechoic lesion (arrows) in the right hepatic lobe. **(B)** This lesion showed peripheral rim-like slight hyperenhancement (arrows) in the arterial phase (17 s). **(C)** The degree of enhancement washed out fast than the surrounding liver parenchymal and displayed hypoenhancement (arrows) in the portal phase (50 s). **(D)** In the late phase (135 s), the lesion remained hypoenhancement (arrows).

**Figure 3:**
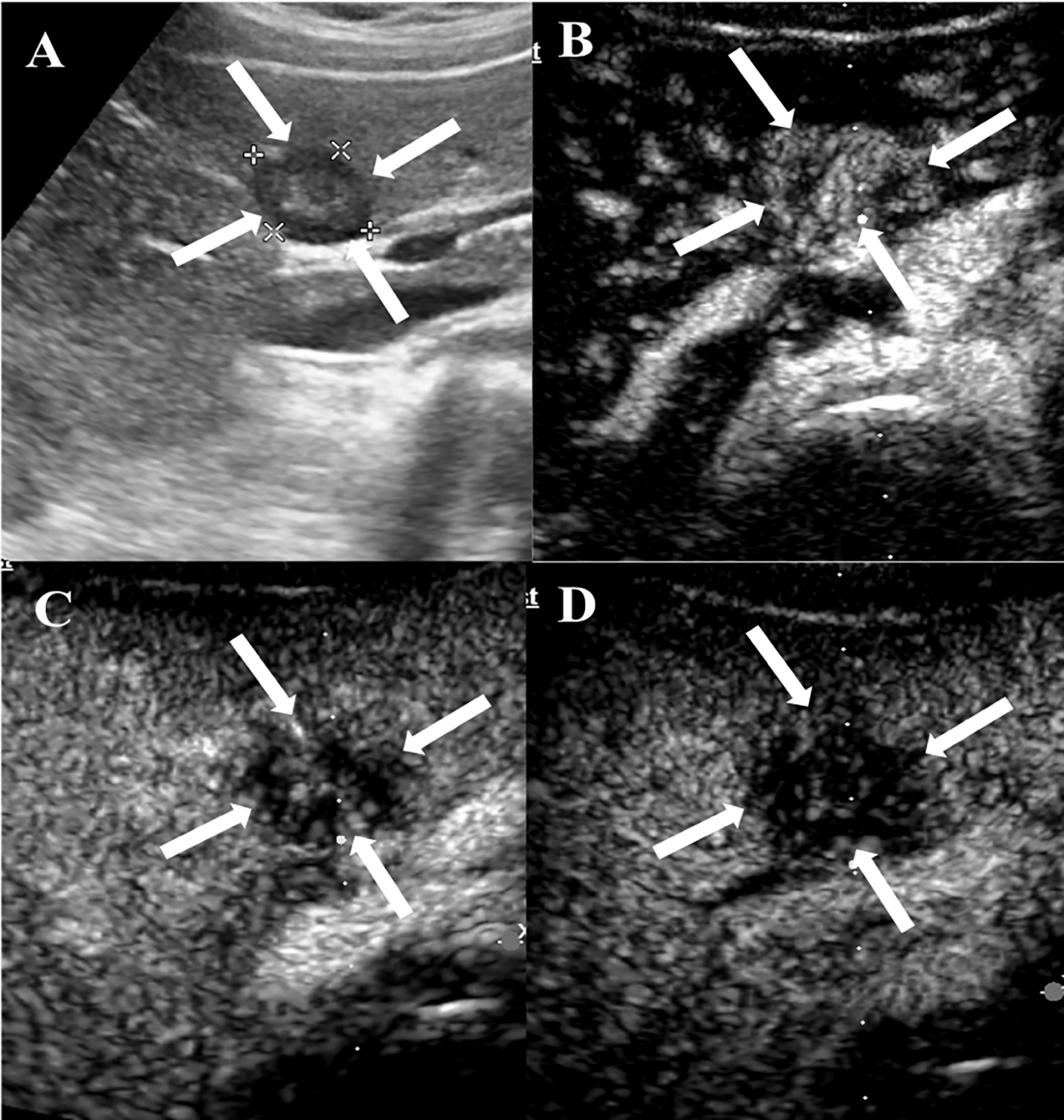
CEUS features of HEHE in a 29-year-old female. **(A)** Greyscale ultrasound illustrated a subcapsular hypoechoic lesion (arrows) in the left hepatic lobe. **(B)** In the arterial phase (12 s), the lesion showed heterogeneous hyperenhancement (arrows) with central unenhanced area. **(C)** The lesion washed out quickly and presented hypoenhancement (arrows) in the portal phase (67 s). **(D)** The lesion demonstrated marked hypoenhancement (arrows) in the late phase (174 s).

**Figure 4:**
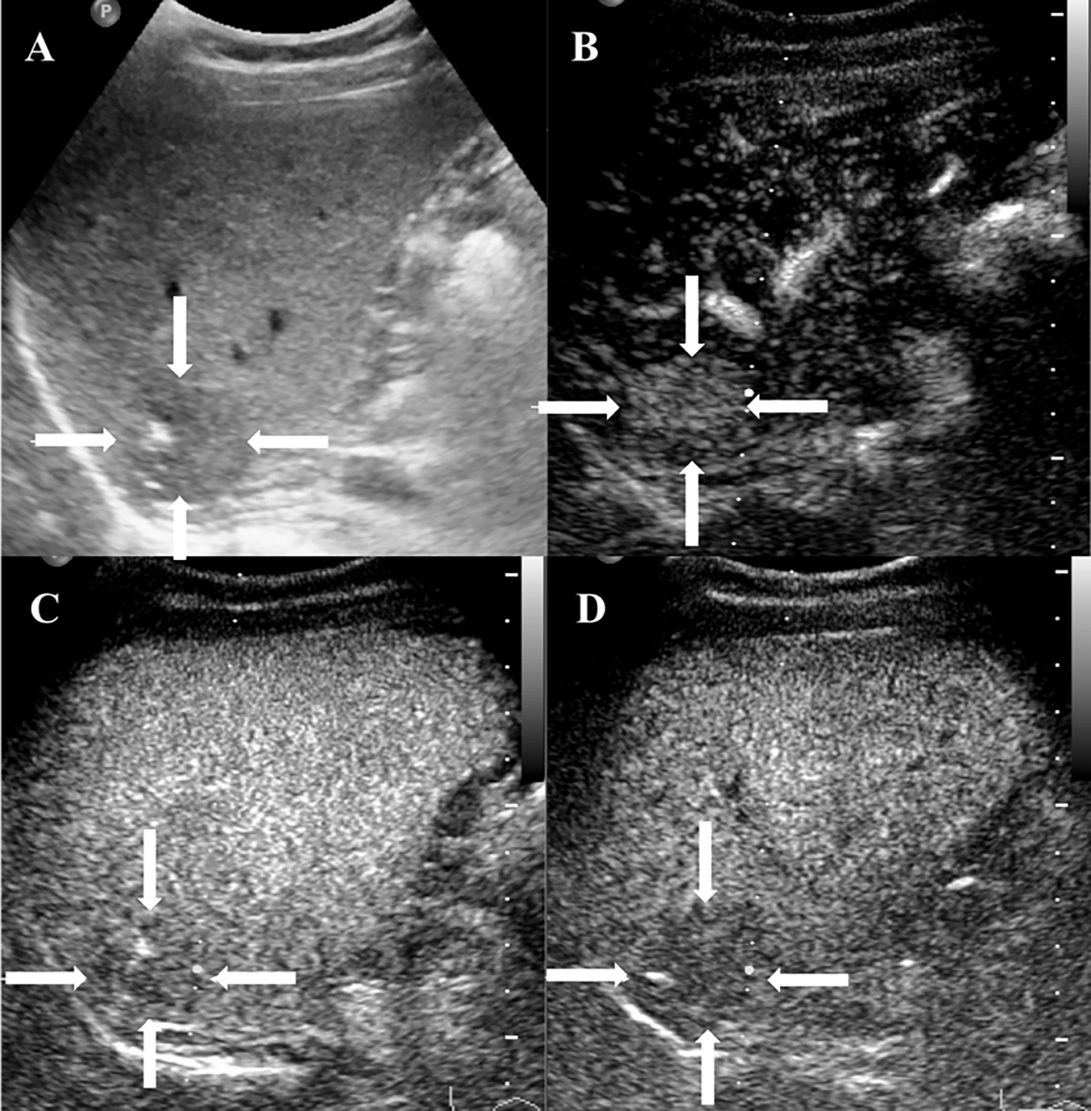
CEUS features of HEHE in a 38-year-old female. **(A)** Grayscale ultrasound displayed a subcapsular hypoechoic lesion (arrows) with ill-defined margin and calcification. **(B)** Homogeneous hyperenhancement (arrows) was seen in the arterial phase (12 s). **(C)** The lesion showed slight hypoenhancement (arrows) in the portal phase (85 s). **(D)** In the late phase (185 s), the lesion presented hypoenhancement (arrows).

Among the 20 patients, eight patients received additional CEUS examination. During the arterial phase, five patients (5/8, 62.5%) showed peripheral rim-like hyperenhancement (**Figure 2**), two patients (2/8, 25%) showed heterogeneous hyperenhancement (**Figure 3**), and one patient (1/8, 12.5%) presented homogeneous mild hyperenhancement (**Figure 4**). All patients (8/8, 100%) showed contrast agent wash-out and presented hypoenhancement in the portal and late phase (**Figures 2**–**4**). In addition, central irregular nonenhancement zones were observed in the lesions of 6 patients (6/8, 75%) at all phases of CEUS (**Figures 2**–**4**). CEUS detected more lesions than CUS in 3 patients (3/8, 37.5%). Using CEUS, five patients (5/8, 62.5%) were misdiagnosed as intrahepatic cholangiocarcinoma (ICC), three patients (3/8, 37.5%) were misdiagnosed as metastases (**Table 3**).

**Table 3:** The CEUS features of HEHE in 8 patients.

CEUS features	
Arterial phase	
Peripheral rim-like hyperenhancement	5 (62.5%)
Heterogeneous hyperenhancement	2 (25%)
Homogeneous hyperenhancement	1 (12.5%)
Portal phase	
Hypoenhancement	8 (100%)
Hyperenhancement	0
Isoenhancement	0
Late phase	
Hypoenhancement	8 (100%)
Hyperenhancement	0
Isoenhancement	0
Irregularly unenhanced zones	
Yes	6 (75%)
No	2 (25%)
Detected more lesions than US	
Yes	3(37.5%)
No	5(62.5%)
Diagnosis with CEUS	
Correct diagnosis	0
Misdiagnosis	8 (100%)

CEUS, contrast enhanced ultrasound; HEHE, hepatic epithelioid hemangioendothelioma; US, ultrasound.

### Contrast-Enhanced CT/MRI Findings

Among the 20 patients, eighteen patients underwent contrast-enhanced CT or MRI examination, including fifteen contrast-enhanced CT and twelve contrast-enhanced MRI.

**Figure 5:**
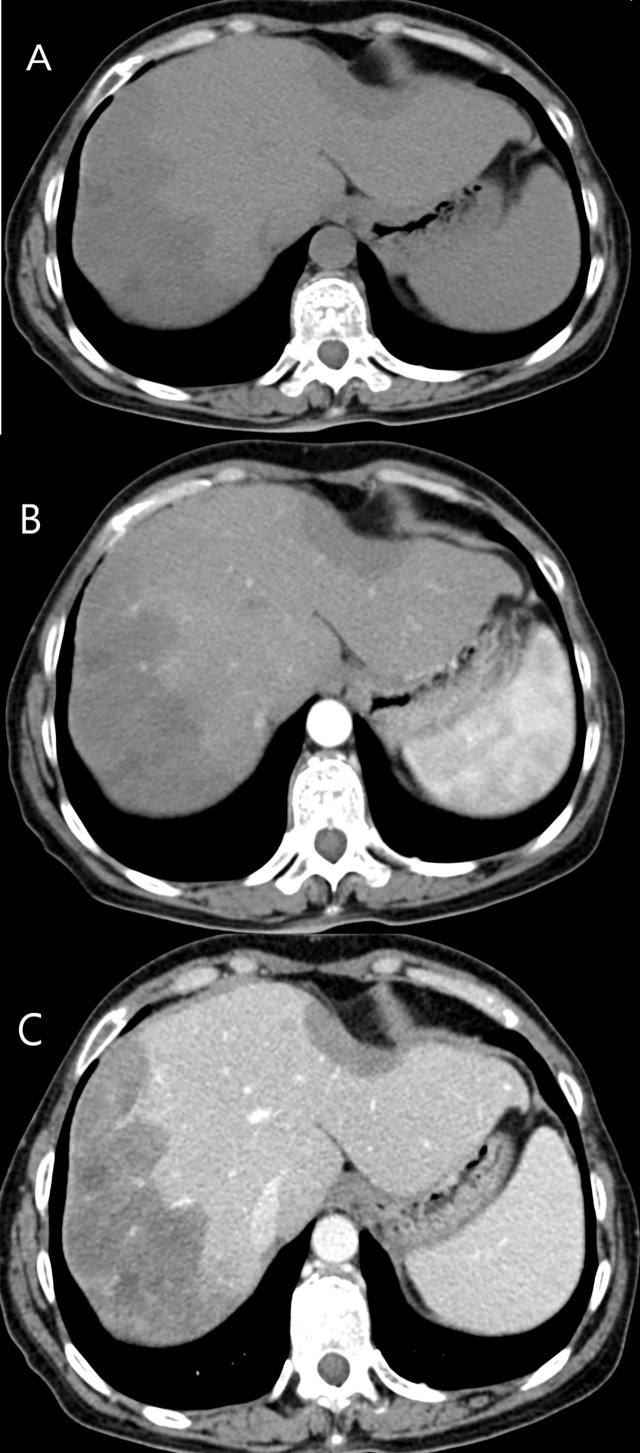
CT features of HEHE in a 43-year-old male. **(A)** Plain CT revealed two low density lesions, accompanied by the capsule retraction sign. **(B)** Mild enhancement during the arterial phase was illustrated. **(C)** Both of lesions revealed hypoenhancement in the portal phase. Lollipop sign was showed more obvious after contrast agent injection.

Among 15 patients who received contrast-enhanced CT examination, ten patients (10/15, 66.7%) showed mild enhancement during the arterial phase, and hypoenhancement in the portal and late phase (**Figure 5**). Five patients (5/15, 33.3%) showed rim-like enhancement in the arterial phase, and progressive centripetal fill-in enhancement in the portal and late phase. Twelve patients (12/15, 80%) revealed lollipop sign (**Figure 5**).

**Figure 6:**
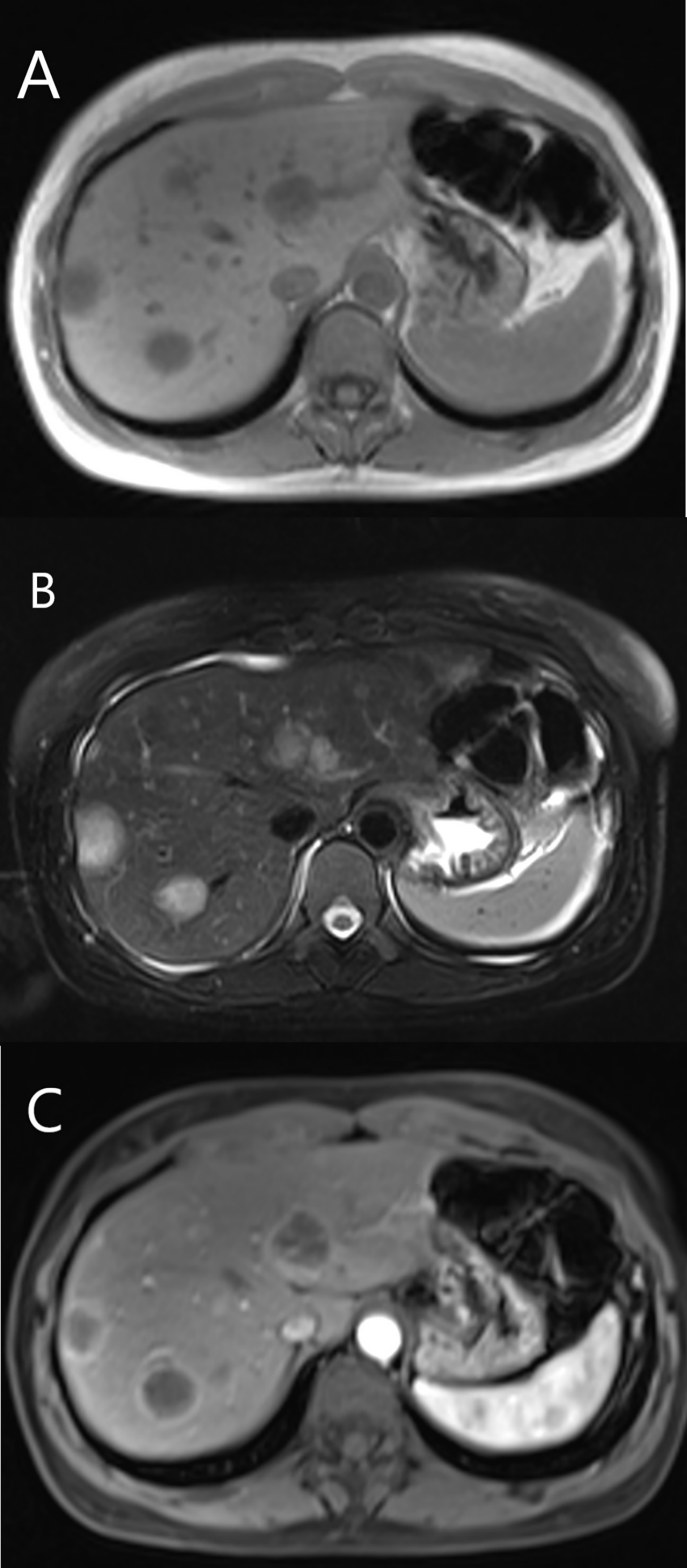
MRI features of HEHE in a 35-year-old female. **(A)** Hypointense showed on T1-weighted image. **(B)** The lesions displayed hyperintense on T2-weighted image. **(C)** Rim-like enhancement and lollipop sign were seen after contrast agent injection.

Among 12 patients who received contrast-enhanced MRI examination, six patients (6/12, 50%) showed mild enhancement in the arterial, portal and late phase. Five patients (5/12, 41.7%) showed rim-like enhancement (**Figure 6**), and four of them showed progressive centripetal fill-in enhancement, one of them revealed continuous enhancement in the portal and late phase. One patient (1/12, 8.3%) showed hyperenhancement in the arterial phase, continuous enhancement in the portal and late phase. Ten patients (10/12, 83.3%) revealed lollipop sign (**Figure 6**).

### Pathological Features

**Figure 7:**
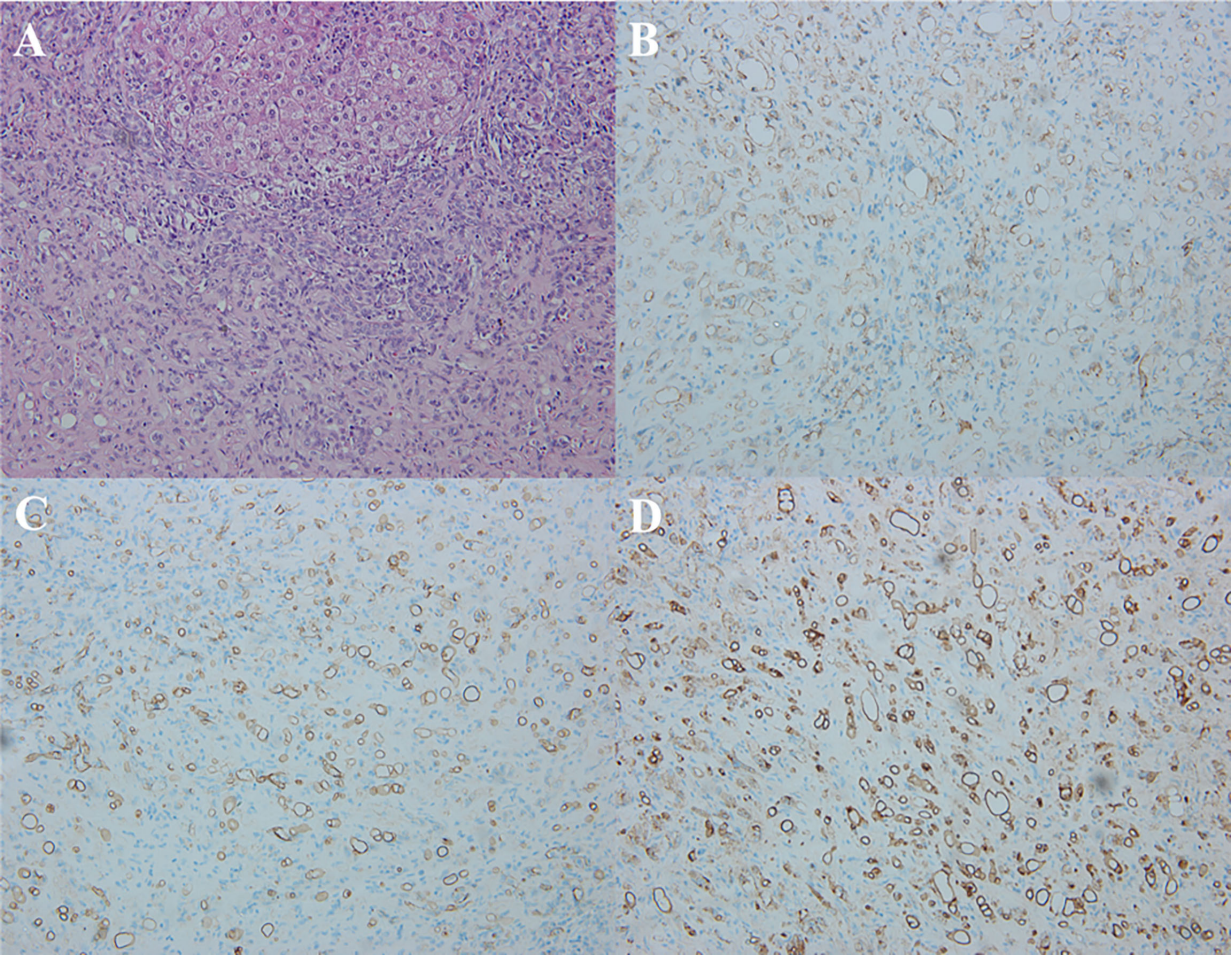
Pathological features of HEHE. **(A)** Microscopy showed the signet ring-like appearance in the tumor epithelioid cells with intracytoplasmic lumina, containing small amount of red blood cells (H&E staining, × 200). **(B)** Immunohistochemical staining revealed positive CD31 in the tumor (×200). **(C)** Immunohistochemical staining revealed positive CD34 in the tumor (× 200). **(D)** Immunohistochemical staining revealed positive FVIII Ag in the tumor (×200).

Histologically, H&E staining showed that HEHE consisted of a central dense stroma with large amounts of mucus, and a peripherally rich cellular area. The signet ring-like appearance were found in the epithelioid cells with intracytoplasmic lumina, sometimes containing red blood cells (**Figure 7A**). Immunohistochemical staining expressed positively for the endothelial markers in the HEHE, which included CD31 (**Figure 7B**) in 19 patients (19/20, 95%), CD34 (**Figure 7C**) in all patients (20, 100%), and FVIII Ag (**Figure 7D**) in 16 patients (16/20, 80%).

## Discussion

HEHE is a rare vascular neoplasm that arises in the liver. It is characterized by the presence of epithelioid endothelial cells. The etiologic factors and mechanism of HEHE remain unclear, but several risk factors have been proposed in the pathogenesis of HEHE. These include oral contraceptives, liver trauma, hepatitis virus, and vinyl chloride (2, 3). Four patients in our study were HBsAg positive, which is similar to the incidence described in previous studies. However, the relationship between hepatitis B virus and the occurrence of HEHE needs to be further elucidated with more studies (14). HEHE generally affects adults with a higher prevalence in females. The mean age at presentation is about 42 years (peak incidence age: 30-50 years), and the ratio of male to female is 1:1.5 (3, 5, 8). Our study included 8 males and 12 females with a mean age of 43.6 years (range 23-69 years), which were comparable with previous studies.

The clinical manifestations of HEHE are variable and nonspecific, and 22-25% of patients are detected incidentally (3). The laboratory parameters are also nonspecific, with mild elevated liver function tests as the most common presentation (6). Serum tumor markers levels are typically within normal ranges. Elevated CEA levels have been documented in a small number of reported cases and are considered to have no clinical value for the diagnosis of HEHE (3, 11). However, in this study, the tumor markers including AFP, CEA, CA 19-9, and CA 125 were elevated in 9 patients. The reasons for this difference are unclear for us.

At present, less than 500 patients of HEHE have been reported in the literature (15). However, CUS and CEUS features of HEHE have not been well summarized. In our current study, most of HEHE presented as subcapsular (85%), multifocal (60%), and hypoechoic (75%) lesions with regular shape (90%) and well-defined margins (55%). Occasionally a hyperechoic or hypoechoic halo (20%) and calcifications (15%) were also present. Other studies in the literature have reported that HEHE lesions were mostly irregularly shaped with ill-defined margins (9, 10). The reason for this difference is not clear to us. The lesions of HEHE are more commonly located in the right hepatic lobe, especially for solitary lesions (16, 17). The echogenicity patterns of HEHE may be associated with the proportion of peripheral tumor cells and central dense stroma (6). The central dense stroma gradually undergoes hemorrhage, necrosis, fibrosis and occasionally forms calcifications. Focal calcifications on CT have been reported in 20% patients (14). Our study had similar findings, with 15% of patients showing imaging evidence of calcifications. A peripheral halo has been described in different studies utilizing CT, MRI and US (6–9, 14). Schweitzer N et al. found that about 3 of 7 (42.9%) patients displayed the peripheral halo on US (9), Chen et al. reported that 24.3% of the patients showed the halo on CT (6). We had similar findings in our study, with 4 of 20 (20%) patients presenting lesions with halo. Tumor infiltration and occlusion of hepatic sinusoids and small vessels can cause a narrow low-vascular area between the tumor and liver parenchymal, which may explain the halo finding associated with HEHE on US (6, 18). HEHE has been divided into three subtypes including a solitary nodule or mass, multiple lesions, and a diffuse subtype (16). Over time multifocal lesions may gradually enlarge and coalesce forming confluent lesions typically in a subcapsular or peripheral distribution (19). In our study, the majority of lesions were subcapsular growth in 85% of patients.

Eight out of 20 patients received CEUS examination in this study. In the arterial phase, CEUS showed peripheral rim-like hyperenhancement in 62.5% of patients, and homogeneous or heterogeneous hyperenhancement were observed in 37.5% of patients. Washout was observed in all patients during the portal and late phases, which is typical of malignant hepatic lesions. CEUS was able to detect more lesions compared to CUS. These findings were similar with other CEUS studies (10). Peripheral rim enhancement on contrast-enhanced CT or MRI images was revealed in 38.9% (7/18) of our study patients, and CEUS can also detect the rim enhancement pattern in 62.5% (5/8) HEHE patients, which may be used to assist the diagnosis of HEHE (8, 10, 11). These features of HEHE may be associated with its distinctive histological characteristics (16, 20). HEHE is composed of dendritic and epithelioid cells with intracytoplasmic vascular lumina containing blood cells. The peripherally rich tumor cellular proliferation remains active with numerous arterial-venous shunts, which could account for the peripherally rim-like hyperenhancement at arterial phase and wash-out with hypoenhancement at portal and delay phase (18). The proportion of peripheral tumor cells with central dense stroma is variable. With tumor growth, the central dense stroma degenerates gradually with necrosis, fibrosis and reduced blood supply (14). In our study, a central irregular non-enhancing region throughout all phases of contrast enhancement was detected in 75% of patients.

In conclusion, our study shows that CUS and CEUS demonstrated specific findings for HEHE, which includes multifocal hypoechoic lesions in a subcapsular distribution with typical enhancement characteristics of malignant hepatic tumors. These lesions may occasionally show a halo and calcifications. Therefore, when these features are found in mid-aged adults, diagnosis of HEHE should be considered. However, a preoperative biopsy will be required to confirm diagnosis.

## Data Availability Statement

The original contributions presented in the study are included in the article/supplementary material. Further inquiries can be directed to the corresponding author.

## Ethics Statement

The studies involving human participants were reviewed and approved by Ethical Committee of West China Hospital of Sichuan University. The patients/participants provided their written informed consent to participate in this study.

## Author Contributions

TQ, DZ and RF wrote the main manuscript text as co-first author. YL prepared figures and tables. WL reviewed the manuscript. All authors contributed to the article and approved the submitted version.

## Funding

This study is supported by Sichuan Science and Technology Program No. 2020YFS0211 and Post-Doctor Research Project, West China Hospital, Sichuan University Grant No.19HXBH014 and National Natural Science Foundation of China Grant No.82001833.

## Conflict of Interest

The authors declare that the research was conducted in the absence of any commercial or financial relationships that could be construed as a potential conflict of interest.

## Publisher’s Note

All claims expressed in this article are solely those of the authors and do not necessarily represent those of their affiliated organizations, or those of the publisher, the editors and the reviewers. Any product that may be evaluated in this article, or claim that may be made by its manufacturer, is not guaranteed or endorsed by the publisher.
